# Electronic Systems for Patients to Report and Manage Side Effects of Cancer Treatment: Systematic Review

**DOI:** 10.2196/10875

**Published:** 2019-01-24

**Authors:** Lorraine Warrington, Kate Absolom, Mark Conner, Ian Kellar, Beverly Clayton, Michael Ayres, Galina Velikova

**Affiliations:** 1 Section of Patient Centred Outcomes Research Leeds Institute of Cancer and Pathology University of Leeds Leeds United Kingdom; 2 School of Psychology University of Leeds Leeds United Kingdom; 3 Leeds Teaching Hospitals NHS Trust Leeds United Kingdom

**Keywords:** oncology, chemotherapy, patient reported outcomes, patient centered, medical informatics

## Abstract

**Background:**

There has been a dramatic increase in the development of electronic systems to support cancer patients to report and manage side effects of treatment from home. Systems vary in the features they offer to patients, which may affect how patients engage with them and how they improve patient-centered outcomes.

**Objective:**

This review aimed to (1) describe the features and functions of existing electronic symptom reporting systems (eg, symptom monitoring, tailored self-management advice), and (2) explore which features may be associated with patient engagement and patient-centered outcomes.

**Methods:**

The review was registered with the International Prospective Register of Systematic Reviews (PROSPERO) and followed guidelines from the Centre for Reviews and Dissemination (University of York, United Kingdom). Primary searches were undertaken of MEDLINE, Embase, PsycInfo, Web of Science, Cochrane Central Register of Controlled Trials, and the Health Technology Assessment databases. Secondary searches were undertaken by screening reference lists and citations. Two researchers applied broad inclusion criteria to identify and select relevant records. Data were extracted and summarized using Microsoft Excel. In order to meet the aims, the study selection, data extraction, and data synthesis comprised two stages: (1) identifying and characterizing available systems and (2) summarizing data on patient engagement and patient-centered outcomes.

**Results:**

We identified 77 publications relating to 41 distinct systems. In Stage 1, all publications were included (N=77). The features identified that supported clinicians and care were facility for health professionals to remotely access and monitor patient-reported data (24/41, 58%) and function to send alerts to health professionals for severe symptoms (17/41, 41%). Features that supported patients were facility for patients to monitor/review their symptom reports over time (eg, graphs) (19/41, 46%), general patient information about cancer treatment and side effects (17/41, 41%), tailored automated patient advice on symptom management (12/41, 29%), feature for patients to communicate with the health care team (6/41, 15%), and a forum for patients to communicate with one another (4/41, 10%). In Stage 2, only publications that included some data on patient engagement or patient-centered outcomes were included (N=29). A lack of consistency between studies in how engagement was defined, measured, or reported, and a wide range of methods chosen to evaluate systems meant that it was not possible to compare across studies or make conclusions on relationships with system features.

**Conclusions:**

Electronic systems have the potential to help patients manage side effects of cancer treatment, with some evidence to suggest a positive effect on patient-centered outcomes. However, comparison across studies is difficult due to the wide range of assessment tools used. There is a need to develop guidelines for assessing and reporting engagement with systems, and a set of core outcomes for evaluation. We hope that this review will contribute to the field by introducing a taxonomy for characterizing system features.

**Trial Registration:**

PROSPERO CRD42016035915; www.crd.york.ac.uk/PROSPERO/display_record.asp?ID=CRD42016035915

## Introduction

Increased efficacy of cancer treatments has led to a rising global population of people living with and beyond cancer. Effective multimodal cancer treatments can slow disease progression, ease the symptoms of the disease, and in some cases cure disease altogether. However, treatments can cause a vast array of side effects such as nausea, pain, fatigue, and diarrhea, which may negatively affect a patient’s quality of life (QoL) and may even become life-threatening, with severe cases such as neutropenic infections. Many cancer treatments are delivered in an ambulatory setting and methods of follow-up and support are highly variable dependent on disease, treatment type, and local practice and resources. Information is commonly provided by the health care team on expected and possible side effects, and patients are advised to seek help if symptoms become a cause for concern. However, patients may not always be able to fully absorb this information at the time it is provided [1] or feel confident in making decisions on when additional hospital contact is necessary between routine clinical reviews [2]. Furthermore, clinicians are mainly reliant on interpreting patient retrospective reports of treatment side effects to ensure safety of care and manage supportive medications. Side effects are not often documented in medical records in a consistent and comparable way [3].

Over the past decade, there has been a dramatic increase in the number of electronic systems developed to support patients during and after cancer treatment by using patient-reported outcome measures (PROMs) to remotely assess symptoms [4-8]. The routine use of PROMs in oncology care as a strategy to enhance symptom monitoring has demonstrated many benefits, such as improved communication between clinicians and patients, and better symptom awareness [9]. Using electronic systems to collect and manage PROMs data has the potential to overcome some of the common challenges previously associated with collating data collected on paper. More recently developed systems can be accessed from any Web-enabled device, allowing patients to report symptoms from home using their own electronic devices such as computers, tablets, or mobile phones. This can be done in real time, rather than relying on retrospective reporting and potentially allows automated documentation of patient reports in the medical record [10].

There is considerable variation in the features offered by symptom reporting systems. Some primarily focus on making symptom data routinely available to health professionals and provide alerts when severe symptoms have been reported [5,11-15]. Others have been developed with a greater focus on patient self-management, delivering tailored and automated self-management advice when appropriate, and advising patients to contact their health care team when necessary [8,16-20]. Some systems use a combination of both approaches [4] and can also include additional features such as facilitating communication with medical teams or other patients.

The availability or absence of certain features may affect how patients engage with systems [21,22]. The terms “engagement” and “adherence” are often used interchangeably in this context. However, adherence suggests an optimal way to use a technology and this is not always easy to define [23]. For the purposes of this review, we refer to engagement in a broad sense of levels of patient usage of the technology. Understanding the key components that can enhance patient engagement with electronic symptom reporting is potentially crucial for improving the development of future systems and encouraging their implementation into standard practice. There are many factors that are likely to have an impact, from individual differences [24], socioeconomic status and healthy literacy [25], to basic system usability [21,26]. There is relatively little currently known about the underlying processes and particularly the role that the availability of systems features might play. However, there is evidence to suggest that individuals vary in the features that they value and use most [20]. In addition, needs may change over time, as patients become more experienced with the system, but also with their disease and treatment [27].

The presence or absence of system features is also likely to affect the level of patient benefit gained from using the system. For example, changes in behavior or disease outcome have been more often observed with interactive interventions in comparison with those that are purely educational [28]. While the use of interactive online systems is associated with greater self-efficacy, better self-management, and more participation in health care [29-32], this may be associated only with specific features such as interactive communication and progress tracking features [33], and consultation and self-management support [34].

Systematic reviews traditionally focus on high-quality evidence for a specific research question. However, increasingly, the value of taking a broader approach to inclusion is being recognized as important to answer complex research questions, particularly in the emerging field of online health interventions [35,36]. With this in mind, the focus of this review was to take an inclusive approach to systematically review and describe the features and functions of existing systems. We also wanted to focus on understanding the level of evidence indicating whether key system features are associated with better patient system engagement and patient-centered outcomes.

The aims of this systematic review are to (1) describe the features and functions of existing electronic symptom reporting systems developed for patients during cancer treatment, and (2) explore which features of these systems may be associated with patient engagement and outcomes. Specifically, we wanted to summarize (1) patient engagement and whether this is related to specific system features (eg, symptom monitoring, tailored self-management advice), and (2) patient-centered outcomes used to evaluate systems and whether better outcomes are associated with specific features.

## Methods

### Protocol and Registration

Details of the protocol were registered on the International Prospective Register of Systematic Reviews (PROSPERO) database [37]. There were no major deviations from the protocol. However, study selection, data extraction, and data synthesis comprised two stages: (1) identifying and characterizing available systems, and (2) summarizing data on patient engagement and patient-centered outcomes. This staged approach was not initially planned but was necessary in order to meet the aims of the review.

### Eligibility Criteria

The review question was refined using Population, Intervention, Comparator, Outcomes, Study design (PICOS) criteria (Table 1), and eligibility criteria were developed based on this. For Stage 1, we wanted an overview of all systems available, so all relevant publications including published abstracts, protocols, and qualitative studies were included. However, discussion papers or systematic reviews were excluded. For Stage 2, in order to review evidence available on patient engagement and any patient-centered outcomes, we wanted to include feasibility studies with any evaluation data of patient use, rather than restricting criteria to randomized controlled trials (RCTs) only. Only full papers were included in this stage. Criteria were piloted by 2 researchers (LW and KA) on a subset of 10 randomly selected papers and subsequently refined and clarified before the next stage.

### Information Sources

Studies were identified from systematic searches of Medline, Embase, PsycInfo, Web of Science, Cochrane Central Register of Controlled Trials, and the Health Technology Assessment databases in March 2016. Due to the nature of the review, results were limited to those published after 2000. No restrictions were imposed on language of publication. Searches were updated on September 12, 2017. Reference lists of relevant publications were screened to identify papers not picked up by the electronic searches. In addition, citations of selected key papers were searched.

### Search Strategy

A detailed example of the search strategy used for Medline is outlined in Textbox 1. This search strategy was adapted for each of the databases.

### Study Selection

For initial screening, a decision for inclusion was made based on title and where available, abstract. This was carried out by one researcher (LW) only, and for this reason, a cautious approach erring on the side of over-inclusion was used. Following this, 2 researchers (LW and KA) independently assessed all remaining papers for relevance. Disagreements were resolved by consensus after referring to the protocol. All discussions and decision making were documented. Where there was insufficient information to make a decision, authors were contacted for further information. If no response was received within 2 weeks, a final decision was made based on available information.

**Table 1:** PICOS (Population, Intervention, Comparator, Outcomes, Study) criteria.

Category	Criteria
Population	Male and female adults >18, no upper age limit, worldwide with any cancer diagnosis, receiving cancer treatment OR within 3 months of completing treatment. The cancer treatment to include any treatment with significant side effects (eg, systemic therapies, radiotherapy, biological therapies).
Intervention	Online systems for patients to report or manage symptoms and side effects during cancer treatment from home; Internet-based or -enabled systems, including mobile apps. Other forms of interactive health communication applications, eg DVDs, games were excluded. Purely educational systems not interactive in any way were excluded. Systems developed to assess and monitor purely psychosocial symptoms were excluded (eg, depression, anxiety, emotional coping or stress). Sleep and fatigue were included. Systems designed to be accessed at one time point only were excluded; access to the system had to be ongoing.
Comparator	Stage 2 only: The review included studies with any comparator (eg, randomized or nonrandomized studies), in addition to studies with no comparator (eg, feasibility studies).
Outcomes	Stage 1: Dependent on the nature and number of papers found, we aimed to characterize systems. For example, we identified if studies included features such as Monitoring of symptoms by health care professionals (HCPs), Alerts for severe symptoms sent to HCPs, Monitoring of symptoms by patients (eg, graphical or tabular), Automated feedback/advice based on responses, Access to symptom information, Communication with other cancer patients, Direct communication with HCPs (distinct from symptom monitoring by HCPs). Stage 2: We aimed to collect where available, information on engagement with systems and information on any patient-centered outcomes, including but not restricted to any QoL measures; self-efficacy measures including patient activation, patient empowerment, mastery; and patient satisfaction.
Study design	Stage 2 only: The review was not restricted to randomized controlled trials, and feasibility studies with any evaluation data were included. Patients had to be using the system over time, and there had to be at least one intended time point of use more than 3 weeks after baseline. This timeframe was selected as many standard chemotherapy treatments are administered every 3 weeks.

**Example of search strategy used (Ovid Medline).**

1. Neoplasms/
2. oncolog*.mp.
3. cancer patient*.mp.
4. 1 or 2 or 3
5. Medical Informatics/
6. Telemedicine/
7. Mobile Applications/
8. Smartphone/
9. Self Report/
10. Self Care/
11. Self-Assessment/
12. (electronic adj2 (Patient report* or Patient-report* or Self report* or Self-report* or Self manage* or Self-manage* or Self monitor* or Self-monitor* or Symptom report* or Symptom-report* or Symptom manage* or Symptom-manage*)).mp.
13. (online adj2 (Patient report* or Patient-report* or Self report* or Self-report* or Self manage* or Self-manage* or Self monitor* or Self-monitor* or Symptom report* or Symptom-report* or Symptom manage* or Symptom-manage*)).mp.
14. (web* adj2 (Patient report* or Patient-report* or Self report* or Self-report* or Self manage* or Self-manage* or Self monitor* or Self-monitor* or Symptom report* or Symptom-report* or Symptom manage* or Symptom-manage*)).mp.
15. (remote* adj2 (Patient report* or Patient-report* or Self report* or Self-report* or Self manage* or Self-manage* or Self monitor* or Self-monitor* or Symptom report* or Symptom-report* or Symptom manage* or Symptom-manage*)).mp.
16. 5 or 6 or 7 or 8 or 9 or 10 or 11 or 12 or 13 or 14 or 15
17. 4 and 16

Limit 17 to (humans and yr=“2000 -Current”)

### Data Items

For Stage 1, basic data were extracted on authors, title, year of publication, and country of origin, in addition to the name (if any given) and type of system being described (eg, Web-based or mobile app). If the system did not already have a descriptive name, an arbitrary name was assigned (eg, System A). A preliminary list of common features was created based on existing knowledge and further developed throughout data extraction until a comprehensive list of common or important features was identified. Data were extracted from each publication on the presence of each feature. This was coded as “Yes” only if it was explicitly described in the publication, otherwise it was coded as a “#” For abstracts, if it was unclear whether or not a feature was present by information available in an abstract, this was classed as “Unable to determine.” Where information was lacking, authors were not contacted for information. However, searches were undertaken for other publications related to the same system.

For Stage 2, data were extracted from studies with some form of system evaluation (eg, patient use of system or evaluation of efficacy). This included data on the number of patient participants, baseline demographics, disease and treatment type, duration of the evaluation, methods used to assess engagement, and actual usage or adherence. Where available, data were also extracted on any patient-centered outcomes used and results of evaluation.

### Data Extraction

Data were extracted using the online Systematic Review Data Repository [38]. The form was piloted on 10 randomly selected papers and further refined. For Stage 1, three additional researchers (KA, BC, MA) each double-coded a number of allocated publications, totaling 36% (27/77) of the overall included publications. A high level of agreement (86%) was found. Discrepancies were resolved by referring back to the protocol and additional publications where available. For Stage 2, the same 3 researchers again each double-coded a proportion of the included publications totaling 46% (13/29) and 100% agreement was found.

### Quality Assessment

Quality was assessed using the Downs and Black checklist for nonrandomized studies [39] and was undertaken alongside data extraction. It was deemed appropriate to assess only studies that included some feasibility/evaluation data, that is, publications included in Stage 2. Studies were given a score along a possible range of 0-26.

### Synthesis of Results

A narrative synthesis was undertaken using the guidelines outlined by the Economic and Social Research Council [40]. Microsoft Excel was used to manage data. For Stage 1, information from multiple publications relating to the same systems was pooled to form a description of features. Where information was conflicting due to earlier and later iterations, the most recent description was used. For Stage 2, information was collected on how patient engagement was assessed for any feasibility study or trial that included these data. For trial studies, information was collected on primary and secondary study outcomes and any results recorded. We then summarized these data to explore any relationships with system features identified in Stage 1.

## Results

### Study Selection

**Figure 1:**
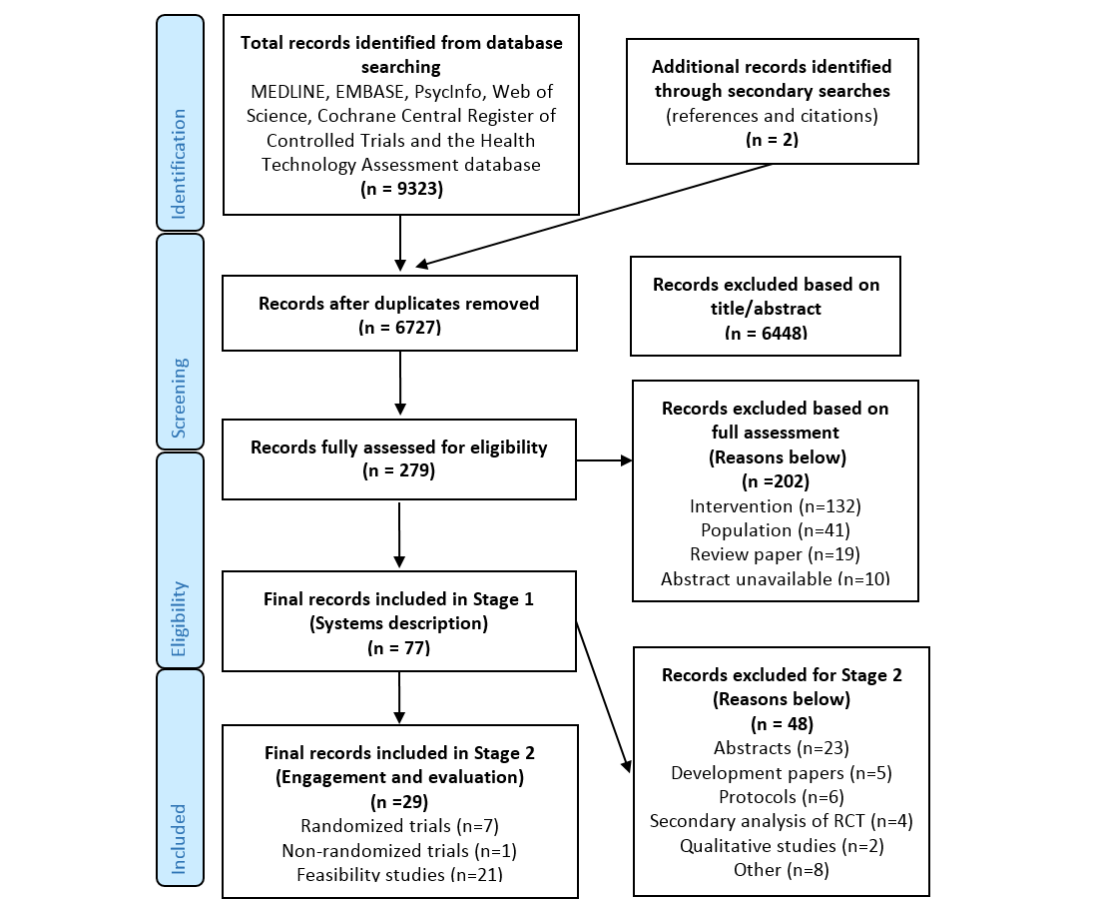
Summary of papers identified and subsequently excluded/included in this review.

An overview of search and selection procedures is outlined in Figure 1. A total of 6727 publications were identified after removal of duplicate publications, including two publications identified from secondary searches (ie, citation and reference lists). All publications were in English. We assessed 279 publications for eligibility, and a total of 202 papers were excluded at this point based on predefined eligibility criteria (intervention, eg, not home-based or Web-based, n=132; population, eg, patients not on active treatment, n=41; discussion paper or systematic review, n=19; or abstract unavailable, n=10). We included 77 publications in Stage 1 of the review (ie, systems descriptions). A large proportion (23/77, 30%) of these publications were abstracts. The reasons for exclusions are outlined in Figure 1. Those 8 publications categorized under “Other” included 2 summary papers giving an overview of development and evidence for a system, a description of standard usability testing, a cost-effectiveness analysis, a content analysis of email communication within a system, a discussion of design approaches and methodology, an evaluation focusing on blood monitoring, and one publication where we were not able to access the full paper and did not receive a response from the authors when this was requested. We identified 29 publications for inclusion in Stage 2 of the review (ie, patient engagement and evaluation of systems). These were 21 feasibility studies and 8 controlled trials (7 randomized and 1 nonrandomized).

### Stage 1: Description of Systems and Features

The 77 publications referred to 41 individual systems. Most originated from the United States (19/41, 46%) or the United Kingdom (6/41, 15%), and all publications were available in English. Systems were commonly Web-based (24/41, 56%), 27% (11/41) were mobile apps, 2 were both mobile and Web-based (2/41, 5%), and 22% (9/41) were Web-enabled mobile devices purposely designed for symptom reporting and were provided to patients for the duration of the study.

**Figure 2:**
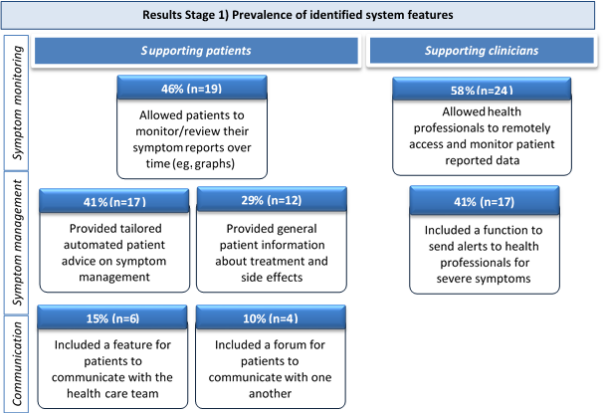
Overall summary of prevalence of identified system features.

Seven common system features were identified. Figure 2 outlines each of the features and its prevalence in the 41 identified systems. Features could be categorized broadly as supporting patients to monitor and manage their own symptoms, and to communicate with health professionals and one another, or supporting clinicians to monitor and manage patient symptoms.

Table 2 [4-8,11-20,41-102] provides an overview of each identified system and its associated publications, in addition to the presence or absence of each of the features identified in Figure 2.

### Stage 2: Patient Engagement and Evaluation

#### Quality Assessment

Along a possible range of 0-26, the overall median quality assessment score of studies using the Downs and Black checklist was 17.0 (mean 16.2, SD 5.3, range 2-24). For trials described in the section on patient-centered outcomes [5,6,8,49,60,79,88,100], the median score was higher at 20.0 (mean 20.4, SD 2.6, range 17-24).

#### Patient Engagement

Table 3 [5,6,8,11-15,42,43,49,60,63,65,68,73-75,79,81,82,84,87, 88,90,92,93,100,101] summarizes data on patient engagement from the 29 included studies (ie, 21 feasibility studies, 7 RCTs, and one non-RCT [88]). All 21 feasibility studies (100%) reported some data on patient engagement, although there was variation in how engagement was defined and measured. Three of the eight trials (38%) did not report any data on patient engagement [6,79,100].

Of the 29 studies, the most common method of assessing engagement was the number of symptom report completions or number of times the system was accessed (12/29, 41%) [15,49,60,63,65,68,74,87,88,90,92]. This was given as an overall figure for the whole sample [15,49,68,90,92], as an average per patient [13,15,65,74,90], or with a breakdown of the variance [63,87]. Nine studies (9/29, 31%) assessed adherence by number of actual completions/accesses in comparison to the number of expected completions/accesses [5,13,14,73,75,81,84,93,101]. This was reported as median or mean adherence of the overall sample for the duration of the study period [2,73,75,81,93,101], or with a breakdown of adherence at different time points [14,84]. Only 2 studies studies (2/29, 7%) categorized patients as users or nonusers dependent on predefined criteria [11,12]. Four studies (4/29, 14%) combined results of patients reporting from home and in clinic [11,13-15]. Not all studies reported on actual usage, and some used evaluation questionnaires with or without semistructured interviews to assess acceptability to patients [42,43,65,82].

Due to the variation in the methods of reporting, it was not possible to determine if there was any overall association between engagement and specific system features.

**Table 2:** Identified systems with description of features and associated publications^a^.

System name (country)and type	Publication type (with relevant references)	Allowed health professional to remotely access and monitor patient reported data	Allowed patients to monitor their symptom reports over time (eg, graphs)	Included a function to send alerts to health professional for severe symptoms	Provided tailored automated patient advice on managing symptoms	Provided general patient info about cancer treatment and side effects	Included a feature for patients to communicate with the health care team	Included a forum for patients to communicate with one another
ASyMs (UK) Mobile device	Randomized trial [6], Secondary analysis of RCT^b^ [41], Feasibility studies [42,43], Abstracts [44-47], Other [48]	✓	x	✓	✓	x	x	x
CASSY (USA) Web-based	Randomized trial [49]	x	✓	x	x	✓	x	✓
CHES (Austria) Web-based	Abstract [50]	–	–	–	–	–	–	–
COPE-CIPN (USA) Web-based	Other [51]	–	–	–	–	–	–	–
CORA (USA) Mobile app	Development paper [52], Protocol [53]	x	✓	x	✓	✓	x	x
eRAPID (UK) Web-based	Protocol [4], Abstracts [54-59]	✓	✓	✓	✓	✓	x	x
eSMART (UK) Mobile device	Protocol [7]	✓	✓	✓	✓	✓	x	x
ESRA-C (USA) Web-based	Randomized trial [60], Secondary analysis of RCT [61], Qualitative paper [62]	x	✓	x	✓	x	x	x
Healthweaver (USA) Web-based & mobile app	Feasibility study [63], Development paper [64]	x	✓	x	x	✓	x	x
HSM (UK) Mobile device	Feasibility study [65]	✓	x	✓	✓	✓	x	x
ICT-FP7 (France) Mobile device	Abstract [66]	✓	–	–	–	–	–	–
INTERAKTOR (Sweden) Web-based & Mobile app	Protocol [67]	✓	✓	✓	✓	✓	x	x
KAIKU (Finland) Web-based	Feasibility study [68]	✓	x	x	x	x	✓	x
MADELINE (USA) Mobile app	Abstract [69]	–	–	–	–	–	–	✓>
MSKCC WebCore (USA) Web-based	Abstract [70]	–	–	–	–	–	–	–
Onco-TREC (Italy) Mobile app	Development paper [71], Protocol [72]	✓	✓	✓	✓	x	✓	x
PatientViewpoint (USA) Web-based	Feasibility study [73]	✓	✓	✓	x	x	x	x
PaTOS (USA) Web-based	Feasibility study [74]	✓	x	x	x	x	x	x
Pit-a-pit (Korea) Mobile app	Feasibility study [75]	✓	x	x	x	x	x	x
PRISMS (Australia) Mobile device	Protocol [76], Abstract [77]	✓	✓	✓	✓	✓	x	x
PROCDIM (USA) Web-based	Abstract [78]	✓	✓	–	–	–	–	–
QoC Health Inc (Canada) Mobile app	Randomized trial [79], Other [80]	✓	x	✓	x	x	x	x
RemeCoach (Belgium) Mobile device	Feasibility study [81]	x	x	✓	x	x	x	x
SCMS (Singapore) Web-based	Feasibility study [82], Other [83]	✓	x	x	x	✓	✓	x
STAR (USA) Web-based	Randomized trial [5], Feasibility studies [11-15,84]	x	✓	✓	✓	x	x	x
The Health Buddy (R) (USA) Mobile device	Development paper [85]	✓	x	✓	✓	x	x	x
WebChoice (Norway) Web-based	Randomized trial [8], Secondary analysis of RCT [17,18], Qualitative paper [20], Other [16,19]	x	✓	x	✓	✓	✓	✓
WRITE (USA) Web-based	Abstract [86]	✓	–	–	✓	–	–	–
System A (USA) Web-based	Feasibility study [87]	x	x	✓	x	x	x	x
System B (The Netherlands) Web-based	Nonrandomized trial [88], Development paper [89], Feasibility study [90]	✓	✓	✓	x	✓	✓	✓
System C (USA) Web-based	Other [91]	–	–	–	–	–	–	–
System D (Sweden) Mobile app	Feasibility study [92]	✓	✓	✓	✓	✓	x	x
System E (UK) Mobile device	Feasibility study [93]	✓	✓	✓	✓	x	x	x
System F (Canada) Web-based	Abstract [94,95]	–	✓	–	✓	–	✓	–
System G (Denmark) Web-based	Abstract [96]	–	✓	–	✓	–	–	–
System H (UK) Mobile device	Other [97]	✓	x	✓	x	x	x	x
System I (USA) Web-based	Abstract [98]	–	–	–	–	–	–	–
System J (USA) Web-based	Abstract [99]	✓	–	–	–	–	–	–
System K (Switzerland) Mobile app	Randomized trial [100]	✓	✓	x	x	x	x	x
System L (USA) Mobile app	Feasibility study [101]	✓	x	x	x	x	x	x
System M (USA) Mobile app	Abstract [102]	–	–	–	–	–	–	–

^a^“✓” denotes feature is present, “x” denotes feature is not present, and “–“ denotes that it was not possible to determine whether feature was present or not.

^b^RCT: randomized controlled trial.

#### Patient-Centered Outcomes

All the trials used some measure of patient-centered outcome to evaluate system efficacy, most commonly validated QoL and symptom and psychosocial outcome measures. Table 4 outlines each trial [5,6,8,49,60,79,88,100], the intervention and comparator groups, outcomes reported, and a summary of the results.

##### Global Quality of Life

CASSY [49] and STAR [5] interventions both demonstrated improvements in overall QoL. However, in addition to the online component, CASSY included access to a collaborative care coordinator with experience in cognitive behavioral therapy and psycho-oncology, which is likely to have contributed to the efficacy. In the STAR study, patients were allocated to computer-experienced and inexperienced groups prior to randomization and only the computer-experienced group had access to the system from home. Results are pooled, making it difficult to assess efficacy for our purposes. No significant impact on QoL was found for WebChoice [8].

##### Physical Symptoms

An overall reduction of symptom distress was found in the studies assessing Electronic Self-Report Assessment-Cancer (ESRA-C) [60] and WebChoice [8]. However, in addition to the online intervention, ESRA-C also included a communication coaching component to improve symptom disclosure to physicians. System B [88] was found to have significant positive impact on the general physical complaints subscale compared to the control group.

Advanced Symptom Management System (ASyMs) [6] and Comprehensive Electronic Cancer Support System for the Treatment of Cancer Related Symptoms (CaSSY) [49] both demonstrated positive impact on levels of fatigue while System K [100] demonstrated a lesser decline in functional activity in contrast to the control group, but this was not significant. Both ASyMs and System K were evaluated using the same measure as used to assess symptoms in the intervention, which may have affected results.

##### Self-Efficacy

WebChoice [8] and System B [88] both demonstrated a positive impact on self-efficacy. However, for System B, this was assessed only as a subscale of a main measure. System K [100] reported an improvement in patient empowerment; however, this was assessed using a single item regarding using the Internet for information seeking, which is unlikely to be a reliable measure.

##### Other Psychosocial Outcomes

CASSY [49] and WebChoice [8] demonstrated significant reductions in depression in intervention compared to control groups. System B [88] demonstrated no difference on the depression subscale of a QoL measure but a significant impact on state anxiety and fear related to specific head and neck problems. WebChoice demonstrated no impact on social support [8]. QoC Health Inc [79] was primarily assessed on number of hospital contacts but also included patient scores of convenience and satisfaction using a simple 5-point Likert scale and found an impact for convenience, but not for patient satisfaction.

Due to the considerable variation in outcomes used and study design, it was not possible to assess any relationships between outcomes and system features.

**Table 3:** Overview of patient engagement data.

System name, patient group (patients, N), treatment type and study duration, quality assessment score (QAS)		Method of evaluation/ patient engagement	Brief summary of findings
**Feasibility studies (n=21)**			
	ASyMS-R [42], Lung (N=16) During and 1 month after thoracic radiotherapy QAS=19	Evaluation questionnaire and semistructured interviews	Actual usage not reported
			Patients perceived it to positively impact on care and promote timely reporting and management of symptoms
	ASyMS [43], Colorectal or lung (N=18) During 2 cycles of chemo QAS=15	Evaluation questionnaire	Actual usage not reported
			Patients reported it helped monitor symptoms, promote self-care, and improve symptom management
	HealthWeaver [63], Breast (N=9) Undergoing active treatment, 4 weeks QAS=8	# of completions/ accesses	All patients used website at least 3x/week, 7 patients used it almost daily
			Phone component used almost daily by 5 patients, 3x/week by 1 patient, and 1-2x/week by 3 patients
	HSM [65], Lung or colorectal (N=18) During 2 cycles of chemo QAS=10	# of completions/ accesses and evaluation questionnaires	All patients completed 1-34 symptom reports, average 14 overall (SD 10.2)
			High variation in use of self-management advice
			Patients found system easier to use and more useful than expected
	Kaiku [68], Head & neck (N=5) Radiotherapy, during and 1 month after QAS=12	# of completions/ accesses	514 symptoms reported (including zero grades)
			23 questionnaires completed
			38 messages sent
	PatientViewpoint [73], Breast or prostate (N=47) Medical oncology treatment UTD - 3 onsite visits (not specified) QAS=15	# of accesses/ expected accesses	190/224 symptom reports completed (85%)
			Median expected questionnaires completed by individual patients was 71%
			Majority of questionnaires completed offsite (n=160; 87%)
	PaTOS [74], Any disease site (N=30) Chemo, 10 weeks QAS=6	# of completions/ accesses	28/30 patients observed for 10 weeks
			Total 231 accesses, 193 fully completed
			Total of 1870 symptoms observations (average 69 per patient, 1.5 per day)
	Pit-a-pit [75], Breast (N=30) Neo-adjuvant chemo, 90 days QAS=14	# of accesses/ expected accesses	1215/2700 responses (compliance=45.0 %)
			Median patient-level reporting rate was 41.1% (range 6.7-95.6%)
	RemeCoach [81], Advanced solid tumors, eg, colorectal, gastric-esophageal, and pancreatic adenocarcinoma (N=11) Duration of Teysuno treatment QAS=18	# of accesses/ expected accesses	Average daily compliance 91.2%
			Could not determine longitudinal compliance because of the low patient number using the coach for an acceptable duration of time
	SCMS [82], Breast, lung, or colorectal (N=4) During 4 cycles of chemo QAS=10	Evaluation questionnaire	All patients completed at least 1 symptom report
			Questionnaire revealed patients found system useful and easy to use
	STAR [84], Gynecologic malignancy (N=49) Laparotomy, 6 weeks QAS=20	# of accesses/ expected accesses	Compliance of patients gradually decreased
			92% of patients completed preoperative session, and 74% completed Week 6 session
			Majority of patients (82%) completed at least 4/7 total sessions in STAR
	STAR [11], Gynecologic malignancy (N=80) Chemo, 8 weeks QAS=16	Users/nonusers (logged in/did not log in)	Patients could access from home or in clinic
			25% used only in clinic waiting area, remainder logged in from home and clinic
			Most patients with home computers (83%) logged in from home without reminders
	STAR [12], Not specified (N=180) Chemo, 8 weeks QAS=8	Users/nonusers (logged in/did not log in)	Patients could access from home or clinic
			2/3 voluntarily logged in from home computers without prompting
	STAR [13], Thoracic malignancies (N=107) Chemo, 16 months QAS=20	# of accesses/ expected accesses	Patients could access from home or clinic
			16 patients (15%) accessed system from home
			Home users accessed system more frequently than those using in clinic (avg=23 sessions, range 3-144) vs (avg=9, range 1-36) respectively
	STAR [15], Lung, gynecologic, breast, genitourinary (N=286) Duration of chemo QAS=19	# of completions/ accesses	Patients could access from home or in clinic
			Total 8690 logins (median 17 logins per patient), avg 0.9 logins per patient per week
			71% from home and 29% from clinic
	STAR [14], Gynecologic malignancy (N=96) Laparotomy, preoperatively & weekly 6-wks postlaparotomy QAS=17	# of accesses/ expected accesses	74% (n=71) completed at least 4/7 surveys and were considered responders
			63% (n=69) completed preoperative session. Remaining completed subsequent surveys.
			9 (9%) patients completed only 1 survey
	System A [87], Hepatobiliary and GI (N=20) Preoperatively and 2 weeks after discharge for curative resection QAS=17	# of completions/ accesses	65% (13/20) completed 8 symptom assessments
			75% (15/20) completed 4 QoL assessments
			Mean 7 minutes to complete MD Anderson Symptom Inventory and mean 4 minutes to complete EuroQoL-5D-5L
	System B [90], Head and neck cancer (N=36) Surgery, 6 weeks QAS=17	# of completions/ accesses	All patients used system (total sessions=982)
			Avg no of sessions was 27.3 (SD 18.4, range 4-69)
			Avg session 12 minutes, longest session 1 hour 38 minutes
	System D [92], Prostate (N=9) Radiation, 2 weeks QAS=13	# of completions/ accesses	Patients reported for mean of 10 days
			Estimated time for report 5 minutes
			Self-care advice accessed by 85%, who logged 20 views at 34 symptoms
			59 alerts: 55 yellow and 4 red
	System E [93], Colon (N=6) Complete resection, during 2 cycles of chemo QAS=11	# of accesses / expected accesses	Data entry compliance was excellent (98% of the twice-daily input was complete) from all 6 patients with the exception of one question
	System L [101], Head and neck (N=22) Duration of radiation (approx. 5-7 weeks) QAS=16	# of accesses/ expected accesses	Median compliance 71% (interquartile range [IQR], 45%-80%)
			6 patients (27%) compliance ≥80%, 2 patients (9%) 100% compliant
			Median reports submitted 34 (IQR 21-53)
**Randomized controlled trials (RCTs; n=7; n refers to # of patients expected to use the system [ie, intervention arm])**			
	ASyMS [6], Breast, lung, or colorectal (N=56) 4 cycles of chemo QAS=22	Not reported	Not reported
	CASSY [49], Any cancer diagnosis (N=144) Chemo, radiation, or surgery, 6 months QAS=19	# of completions/ accesses	Total number of page views=1491
			Total duration in minutes=1813.9
			Total views and duration given for individual patients
	ESRA-C [60], Diagnosis of cancer (N=374) Any therapeutic regimen, UTD, over 4 visits QAS=24	# of completions/ accesses	Median access rate of 4 (range 2-4) at study time points
			Median access rates of 1 (range 0-8) at voluntary times
	QoC Health Inc [79], Breast (N=32) Reconstructive surgery, 30 days QAS=23	Not reported	Not reported
	STAR [5], Metastatic breast, genitourinary, gynecologic, or lung (N=286) Duration of chemo QAS=20	# of accesses/ expected accesses	Computer experienced (home access) and inexperienced (clinic access) figures combined
			Avg 73% completed a self-report at any given clinic visit (includes clinic completions)
	WebChoice [8], Breast or prostate (N=162) Surgery plus radiation, chemo, hormone therapy, or a combination, 1 year QAS=23	# of completions/ accesses	77% logged on at least once
			23% never logged on
			Of 103 (64%) who logged on more than once, avg logons=60 times (range 2-892)
	System K [100], Breast cancer (N=95) Adjuvant or neo-adjuvant chemo, 6 weeks QAS=18	Not reported	Not reported
**Non-RCT (n=1) (n refers to # of patients expected to use the system [ie, intervention arm])**			
	System B [88], Head and neck cancer (N=39) Surgery, 6 weeks QAS=17	# of completions/ accesses	Avg # of sessions=27, avg length of session=12 mins
			Avg # of completions=12.6
			Avg # of messages=4.5

**Table 4:** Overview of patient-centered outcomes data.

Study, population (N); study design	Intervention and comparator groups	Outcomes reported	Summary of results
ASyMs [6], Breast, lung, or colorectal (N=112); 2-arm randomized controlled trial (RCT), 4 cycles of chemo	Intervention (N=56): Asked to complete a symptom questionnaire integrating Common Toxicity Criteria Adverse Events (CTCAE) grading system and Chemotherapy Symptom Assessment Scale Symptom information sent in real time to the study server Patients receive severity dependent tailored self-care advice on mobile phone interface Evidence-based risk assessment tool alerts clinicians via a dedicated 24-h pager system of any severe symptoms Comparator (N=56): Standard care following local guidelines and procedures related to monitoring and reporting of chemo-related toxicity including written and verbal information from nurses administering chemo	Primary outcomes: Paper version of online questionnaire; Comparison between groups on mean scores from 4 paper-based completions at baseline and before each chemo cycle	Higher reports of fatigue (*P*=.04) and lower reports of hand-foot syndrome (*P*=.03) in control group compared with intervention group
			No difference on nausea, vomiting, diarrhea, or sore mouth/throat
CASSY [49], Any diagnosis of cancer Chemo, radiation, or surgery (N=261) 2-arm RCT, 6 months	Intervention (N=144): Access to psycho-educational website where patients could record and monitor symptoms via graphs and journal Access chat room to communicate with other study patients Audiovisual and resource library including relaxation techniques and educational videos Phone contact (approx. every 2 weeks) with a collaborative care coordinator with training and experience with cognitive-behavioral therapy and psycho-oncology Comparator (N=117): Usual care provided by medical team plus assessment of symptoms and blood draws at the same time as intervention patients to evaluate efficacy of intervention	Primary outcomes: Depression (Centre for Epidemiologic Studies-Depression≥16), Pain Brief Pain Inventory, Anemia (Functional Assessment of Cancer Therapy [FACT]-Anemia), Hepatobiliary (FACT-Hep)	Reductions of fatigue at 6 months (*P*=.09)
			Statistically and clinically significant changes in overall QoL (*P*=.05)
			Reductions in pain and depression
		Secondary outcomes: Serum cytokines levels and Natural Killer Cell (NK), Comparison at 6 months follow-up	Medium effect size for NK cell number (Phi=0.491) at 6 months (chi-square=3.62, *P*=.057)
ESRA-C [60], Diagnosis of cancer Any therapeutic regimen (N=779) 2-arm RCT, UTD, over 4 visits	Intervention (N=374): Participants completed cancer symptoms and QoL (SxQoL) assessments at each study time point and ad lib between visits Summary reports delivered to clinicians Self-management advice given for 3 symptoms Coaching to verbalize issues to health care team Alert to contact health care team for severe symptoms Patients could monitor symptoms via graphs and journal Self-care strategies and coaching available at any time Comparator (N=378): Participants completed assessments at each study time point Summary reports delivered to clinicians Research staff verbally notified health care team of any severe symptoms reported at clinic visit Both groups were provided the same patient education typically available in each clinic	Primary outcomes: Symptom Distress Scale (SDS) plus 2 items (impact on sexual activity and interest, fever/chills) to form SDS-15, End point was change in SDS-15 total score from baseline to the end-of-study time point	Intervention had lower symptom distress; mean change in SDS-15 score was 1.27 ([SD], 6.7) in control (higher distress) and -0.04 (SD 5.8) in intervention (lower distress)
			SDS-15 score reduced by estimated 1.21 (95% CI 0.23-2.20; *P*=.02) in intervention vs control group
QoC Health Inc [79], Breast cancer Surgery (N=65) 2-arm RCT, 30 days	Intervention (N=32): Follow-up visits at 1 and 4 weeks replaced with examination of surgical site via photos submitted through mobile app, plus completion of pain visual analog scale and quality of recovery 9-item questionnaire Reporting began after discharge from recovery room Email reminder if submission not received Surgeon used wireless interface to access data and monitor patient’s condition Severe scores flagged in the database for quick viewing. Red flags prompted in-person follow-up Physicians summarized data from mobile app using prototypical subjective, objective, assessment, and plan note at 1 or more time points during 30-day monitoring period Comparator (N=33): Patients in conventional follow-up group had planned clinic follow-up at approx. 1 week and 4 weeks after operation	Primary outcomes: Total number of follow-up visits (including specialists, family physician, and emergency department), Total number of phone calls and emails to health care team, Satisfaction and convenience scores using 5-point Likert scale, Postop complications	Control group more likely to attend in-person follow-up care first 30 days after surgery (95% CI 0.24-0.66; *P*<.001)
			Intervention group sent more emails than control group (IRR 4.13; 95% CI 1.55-10.99; *P*=.005)
			Intervention group reported higher convenience scores (IRR 1.39; 95% CI 1.09-1.77; *P*=.008)
STAR [5], Metastatic breast, genitourinary, gynecologic, or lung cancers (N=766) Before randomization, participants assigned to subgroups (computer-experienced and computer-inexperienced) Only computer-experienced intervention used system from home Duration of chemo	Intervention (N=286): Remote access to Web-based interface including questions adapted for patient use from CTCAE Triggered email alerts to nurses when patient-reported symptom worsened by 2 points or reached an absolute grade Report tracking participant’s symptoms printed at each clinic visit for both nurse and treating oncologist No specific guidance provided to clinicians on actions to take in response to alerts or printed symptom profiles Comparators: Intervention – Computer-inexperienced (N=155): Similar to main intervention group but accessed system in clinic only and did not have remote access Computer-experienced – Usual care (N=253) Computer-inexperienced – Usual care (N=72): Usual care for the computer-experienced and computer-inexperienced subgroups consisted of standard procedure for monitoring and documenting symptoms Symptoms discussed and documented in the medical record during clinical encounters between patients and oncologists Patients encouraged to initiate phone contact between visits for concerning symptoms	Primary outcomes: EuroQol EQ-5D Index given via paper at clinic visits every 12 ± 4 weeks throughout study	Combined results for computer-experienced (home system) & computer-inexperienced (clinic only) intervention
		Secondary outcomes: Survival at 1 year, Time to first emergency room visit and time to first hospitalization, Time receiving active cancer treatment, Number of nursing calls to patients	Greater improvement in Health-Related QoL scores in intervention vs usual care arm (34% vs 18%) and worsened among fewer (38% vs 53%; *P*<.001)
			Greater survival in intervention arm (69% vs 75%, *P*=.05)
			Fewer emergency room visits in intervention (34% vs 41%, *P*=.02)
			Intervention received chemo for longer (8.2 vs 6.3 months, *P*=.002)
			No difference in number of nursing calls to patients
WebChoice [8], Breast or prostate cancer Surgery plus additional treatment of either radiation, chemo, hormone therapy, or a combination of those) (N=325) 2-arm RCT, 1 year	Intervention (N=162): Assessment component to monitor and report symptoms, problems, and priorities for support along physical, functional, and psychosocial dimensions Patients receive automated tailored self-management advice based on responses Patients receive advice to contact health care team when appropriate Info can be used to create a self-care plan Info section with access to other reliable, relevant Web resources Communication section including (1) unrestricted support forum for group discussion, allowing patients to post messages anonymously, (2) question-and-answer area where patients can privately ask questions of expert nurses in cancer care Access to diary to keep personal notes Comparator (N=163): In addition to a letter giving their group assignment, participants receive info sheet with suggestions for publicly available, cancer-relevant websites	Primary outcomes: Memorial Symptom Assessment Scale Short Form	Between-group differences significant for the Global Distress Index only (*t*=4.42; *P*=.037)
		Secondary outcomes: Center for Epidemiological Studies Depression scale, Cancer Behaviour Inventory, 15D Health-related QoL, Medical Outcome Study Social Support Survey	No significant differences on the other subscales or total score or any secondary outcomes
			Experimental group showed significant improvements in depression (*t*=-2.71; *P*=.007)
			Control group had worsened self-efficacy (*t*=-2.82; *P*=.005) and Health-related QoL scores significantly (*t*=-2.77; *P*=.006),
System B, Van den Brink [88] Head and neck cancer Surgery (N=163) Nonrandomized trial, 6 weeks	Intervention (N=39): Provided with a laptop Patients could be monitored at home (by means of electronic questionnaires) Could communicate (send messages) to team Access to information Communicate with fellow sufferers (via a forum) Comparator (N=128): Routine follow-up apps at 2 and 6 weeks after discharge Patients could contact their care providers, both in- and outside hospital, if considered necessary	Primary outcomes: QoL measure assessed state anxiety, object anxiety, feelings of depression, uncertainty, feelings of insecurity, loss of control, self-efficacy, loneliness, and complaints	Intervention had significantly better change from baseline at 6 wks for state anxiety (*P*=.01), fear related to specific head and neck problems (*P*=.02), physical self-efficacy (*P*=.03), perceived abilities in swallowing and food intake (*P*=.04), general physical complaints (*P*=.02)
System K, Egbring [100], Breast cancer Adjuvant or neo-adjuvant chemo (N=139) 3 arm RCT, 6 weeks	Intervention (N=49): App and physician: Patients used mobile app and reviewed reported data with treating physician at scheduled visits Patients could report daily functional activity or symptoms with indication of severity Patients could edit a quick list of their preselected symptoms or select any of the 48 symptoms made available from the CTCAE listing Treating physician enabled access to review and discuss electronically reported symptoms during scheduled visits Comparators: Attention-control group (N=46) App only: Patients instructed to use the mobile app without physician review Control group (N=44): Received regular physician support	Primary outcomes: Daily functional activity measured by ECOG	Control groups showed greater decline in functional activity versus intervention but not significant
		Secondary outcomes: Symptom reporting (intervention group and attention control group only), Patient-physician communication (measure not specified), Patient Empowerment (measure not specified)	At last visit, intervention & attention control patients reported fewer concentration issues than control group (*P*=.002)
			At third visit, significantly more intervention & attention control patients confirmed use of Internet for disease information compared vs control

## Discussion

### Principal Findings

The main aim of this review was to systematically describe and assess the features and functions of current systems available for patients to report and manage side effects of cancer treatment. We also wanted to focus on understanding the level of evidence indicating whether key system features are associated with better patient system engagement and patient outcomes.

In Stage 1 of the review, we identified a total of 41 individual systems. There was significant variation between systems, though published descriptions of systems were often limited. We developed a taxonomy of features that were then classified into those supporting clinicians to deliver patient care in an innovative way and those aimed to support patients to better self-manage their condition and identify when medical input may be needed. This was successfully applied to describe the presence or absence of common system features.

The review of features highlighted some interesting findings. It was surprising to note that while over half (58%) of systems had the facility for health care providers to monitor patient data over time, fewer than half (46%) included the facility for patients to monitor and review their own data. Given the available evidence suggesting that self-monitoring is generally beneficial to support patients’ self-management [28,33,103], this feature could be very important to improve efficacy of systems and in most cases, may be relatively easy to implement. Similarly, less than half of the systems (41%) included a feature for delivering advice to support patients to self-manage symptoms and less than a third provided patients with access to general educational information. The two least common features were facilities to support communication between patients and health care providers (15%) and communication between patients themselves, respectively (10%). Previous research has indicated that these features are highly valued and utilized by patients [20,22,29,33]. It is likely that these features are less common due to complexities in their implementation and maintenance. For example, it may be difficult to engage busy clinicians to respond to patient communication in this way, and there are ethical considerations around the need to moderate patient forums that are endorsed by a health care facility.

In Stage 2 of the review, we found little agreement on how patient engagement with systems was defined, measured, or reported, which meant it was not possible to compare levels of engagement across studies or make any conclusions on relationships with system features. Our review also indicated heterogeneity in terms of outcomes used to evaluate systems. Even of those that focused on symptoms or global QoL, the variation in methods and measures used made meaningful comparison impossible.

Due to the heterogeneous nature of reporting engagement and outcomes, we were unable to explore any relationships with system features. Our findings are similar to other reviews undertaken in this area, which have also found that poor assessment and reporting of patient engagement with systems makes comparison between studies difficult. Brower et al made quantifiable and comparable reports of engagement as part of their inclusion criteria for their review, and results indicated that facility for communication with other patients may be a very influential factor in patient engagement and needs careful consideration during system design [22]. However, other oncology specific reviews have found that methods of assessing and reporting patient engagement were too heterogeneous to make meaningful conclusions [104,105]. We identified only 8 trials (7 randomized and 1 nonrandomized) that evaluated systems, none of which reported any analysis on relationships between engagement and outcomes, and 3 of which did not report any data on patient engagement at all. This does not seem to be unique to oncology. Donkin et al [106] set out to review the impact of patient engagement with e-therapies across a range of disease groups and similarly found that this is not a link that is routinely explored.

Robust evidence supporting the value of systems for patient-centered outcomes was limited, with a large proportion of feasibility studies identified and even fewer RCTs. While all trials used some measure of patient-centered outcome to evaluate systems, a wide range of assessment tools were used, again making comparison difficult. In addition, 2 studies used the same measure for symptom assessment as part of the intervention, as for the outcome measure. Only 3 trials reported any measure of self-efficacy or patient empowerment, one of which used a study-specific nonvalidated measure [79], and another that was assessed using a subscale of a global QoL measure [88]. There is an array of evidence to suggest that online interventions can have a positive impact on self-efficacy and patient activation levels [30,32,33,107]. Growing evidence suggests that self-efficacy and patient activation play a significant role in symptom management and quality of life throughout cancer treatment [108,109] and are associated with an array of improved health behaviors and health outcomes [110-112] and lower use of hospital resources [113]. The reviewed systems generally demonstrate positive outcomes for patients as has been found in other reviews [31].

To our knowledge, this is the first systematic review in this field to identify and characterize all available systems for patients to report and manage side effects of cancer treatment, in addition to evidence on patient engagement and patient-centered outcomes.

### Limitations

In order to meet the aims of the review, we included many publications that provided limited information about the system evaluated and some of which were of poor quality. However, we felt that this was necessary in order to meet the aims of the review and evaluate all evidence. Due to limitations on available resources, the initial stage of study selection (ie, assessment of titles and abstracts) was undertaken by a single reviewer. This is a limitation of our methodology and may have resulted in some bias of inclusion. To address this, a cautious approach erring on the side of over-inclusion was adopted, in order for records to be fully assessed by 2 researchers in the next stage of the review.

Due to the heterogeneous nature of study designs and methods of reporting engagement and outcomes, we were unable to explore any relationships with system features. This is a field of research that is still in its infancy, and the large number of feasibility studies and abstracts identified are likely to be indicative of this. The search was last updated September 2017. Due to the fast-moving nature of this field of research, it is likely that additional publications will be available by the time of publication. This is a common limitation of systematic reviews that is particularly pertinent with reviews of technology [114]. We did identify a number of protocols for planned quality trials that may contribute to a more in-depth understanding of associations between system features, adherence, and outcomes in the future [4,7,53,67,72,76]. In addition, we have not explored how issues with implementing systems into clinical practice may have affected the efficacy of systems. A discussion of these issues is outside the scope of this review but has been well-documented elsewhere [115].

### Conclusions

There is a real need for evidence-based guidance on developing, evaluating, and reporting systems. Based on this systematic review, we propose a taxonomy for characterizing system features to guide future development, improvement, and implementation of such systems. More work is needed to develop guidance for standardized reporting of patient engagement both in feasibility studies, and in evaluation trials. This is a complex and multifaceted issue, and it is important that barriers and facilitators to engagement are shared to help the evolution of more sustainable and valuable systems. Similarly, the development of guidance for the evaluation of systems is necessary. Variation in approaches to design and implementation will rightly affect outcomes chosen to evaluate efficacy [104,105]. However, there is enough commonality between systems to call for a set of recommended core outcomes to be developed [116]. More work is needed to develop this, and this is something we will work towards in the future. However, based on this review we recommend that all system evaluations include (1) a description of the system using our taxonomy of system features, (2) measures of feasibility and engagement, (3) patient-centered outcomes focusing on QoL and symptom improvement, in addition to those focusing on self-efficacy and patient activation, and (4) a measure of health economics. This will facilitate synthesis of evidence in order to improve the design of systems and make them practically useful for both patients and clinicians.

## Abbreviations

ASyMs: Advanced Symptom Management System
CASSY: Comprehensive Electronic Cancer Support System for the Treatment of Cancer Related Symptoms
CTCAE: Common Toxicity Criteria Adverse Events
ESRA-C: Electronic Self-Report Assessment-Cancer
HCP: health care professional
PICOS: Population, Intervention, Comparator, Outcomes, Study design
PROM: patient-reported outcome measure
PROSPERO: International Prospective Register of Systematic Reviews
QAS: Quality Assessment Score
QoL: quality of life
RCT: randomized controlled trial
